# Advances in Flame Retardant Poly(Lactic Acid)

**DOI:** 10.3390/polym10080876

**Published:** 2018-08-06

**Authors:** Benjamin Tawiah, Bin Yu, Bin Fei

**Affiliations:** Institute of Textile and Clothing (ITC), The Hong Kong Polytechnic University Hung Hom, Kowloon, Hong Kong, China; benjamin.tawiah@connect.polyu.hk (B.T.); yubin2-c@my.cityu.edu.hk (B.Y.)

**Keywords:** flame retardants, poly(lactic acid) (PLA), 2D nanomaterials, 1D nanomaterials, metals oxide fillers, phosphorus fillers, mechanical properties

## Abstract

PLA has become a commodity polymer with wide applications in a number of fields. However, its high flammability with the tendency to flow in fire has limited its viability as a perfect replacement for the petrochemically-engineered plastics. Traditional flame retardants, which may be incorporated into PLA without severely degrading the mechanical properties, are the organo-halogen compounds. Meanwhile, these compounds tend to bioaccumulate and pose a risk to flora and fauna due to their restricted use. Research into PLA flame retardants has largely focused on organic and inorganic compounds for the past few years. Meanwhile, the renewed interest in the development of environmentally sustainable flame retardants (FRs) for PLA has increased significantly in a bid to maintain the integrity of the polymer. A review on the development of new flame retardants for PLA is presented herein. The focus is on metal oxides, phosphorus-based systems, 2D and 1D nanomaterials, hyperbranched polymers, and their combinations, which have been applied for flame retarding PLA are discussed. The paper also reviews briefly the correlation between FR loadings and efficiency for various FR systems, and their effects on processing and mechanical properties.

## 1. Introduction

The increasing use of polymer materials in everyday life is driven by their remarkable combination of properties such as light weight, ease of processing and cost efficiency [1,2]. With the recent awareness of the effect of synthetic polymers on the environment coupled with the stringent environmental regulations by governments across the globe; the quest for alternative bio-based (biodegradable) polymers has increased significantly [3]. In recent times, the world production of bio-based polymers has seen a remarkable growth of 4% to 6.6 million tons from 2015 to 2016 [4]. This trend has been suggested to continue until 2021 [4]. Out of the myriads of bio-based polymers, the focus has been on those with biodegradable properties such as polyhydroxyalkanoates (PHA), polylactic acid (PLA) and starch blends [4,5]. Meanwhile, PLA has attracted much attention due to its unique properties like good appearance, high mechanical strength, biodegradability and low toxicity [6]. These properties have broadened its applications with an annual projected growth of 10% [4]. The interest in PLA is spurred by recent applications in the automotive industry, building, and construction, electrical and electronics, furnishing, industrial carpets, high-end fashion products, foams, fiberfill, etc. [7]. However, PLA is known for its low melting point and high flammability, sometimes accompanied by the production of toxic gases during combustion in vitiated atmospheres [8]. Consequently, improving the flame retardant (FR) properties of PLA has become a necessity for extending its applications; although a great deal of progress has been made [9,10,11].

Just like any other polymer, the flammability of PLA polymer is defined by well-known parameters, such as burning rate (solid degradation rate and heat release rate), ignition characteristics (delay time, ignition temperature, critical heat flux for ignition), product distribution (particularly toxic species emissions), smoke production [8], etc. The most efficient FR for PLA have been those with the halogen moieties because they have proved largely successful in most polymers [12], but these FRs are recognized as major potential global contaminants due to their adverse health effects in animals and humans [12,13,14]. The halogenated FR leach to the environment throughout the life cycle of treated objects as dust in indoor exposure, mechanical recycling of plastics and metals as well as incineration and open burning of household wastes, such as electronic parts, paints, solvents, and textiles [15,16]. While most halogenated flame retardants are classified as toxic, they also act as potent precursors for the formation of polybrominated dibenzo-*p*-dioxins, dibenzofurans and other radical chemicals species that are dangerous to flora and fauna [12,16,17]. They are particularly associated with such deformities such as endocrine and thyroid disruption, immune toxicity, reproductive defects, cancer, neonatal and fetal deformities, child development and neurologic functions [12,14,18,19,20]. As a result, the halogenated flame retardants are being phased out gradually due to strict government policies and general environmental awareness by consumers [12,21]. The quest for alternative flame retardant has been met with a lot of interesting results from the research community. Yet, no commercial flame retardant has been especially tailored to control the flammability of PLA to preserve its biological integrity while improving or maintaining its subtle crystalline and mechanical properties [22]. This review presents the recent advances in flame-retardant PLA additives with the main focus on other additives besides the pure bio-based systems reviewed extensively [23]. The review also provides insight into FR loading and its correlation with important fire safety parameters such as limiting oxygen index (LOI), vertical burning test (UL-94), peak heat release rate (PHRR), etc., as well as their effect on mechanical properties. Important FR additives such as metal oxides, phosphorus-based fillers, and their combinations, particularly with nitrogen-containing compounds, polymeric FRs, 2D and 1D nanomaterials for PLA has been reviewed.

## 2. Current Trends in Flame-Retardant PLA

### 2.1. Metal Oxide Fillers

The commercially available FR market is mainly composed of alumina/magnesium hydroxides and antimony trioxide [8]. The metal oxides function in both the condensed and gas phase of flames by absorbing heat and decomposing to release water of hydration, especially in the case of antimony trioxide [24,25]. This process may include cooling both the polymer and the flame and diluting the flammable gas mixture. However, very high concentrations (50 to 80 wt %) are required to impart flame retardancy which often affects the mechanical properties of polymers adversely [26]. Alumina trihydrate (ATH) decompose when exposed to temperatures over 200 °C and, therefore, limits its application in polymers that require higher processing temperatures. Magnesium hydroxide, however, is stable to temperatures above 300 °C and can be melt processed into several polymers [25]. Antimony is a metalloid element that has characteristics of both metals and non-metals and although it is not categorized as a flame retardant per se, it is often used as a synergist in plastics, rubbers, textiles, paper, and paints, typically by 2–10 wt %, with other compounds to reduce the flammability of a wide range of plastics and textiles [26,27,28]. The FR mechanism of antimony oxides and antimonates involve conversion to volatile species in fire, which release halogen acids to prevent further spread of fire [29]. The application of certain metal oxides for flame-retardant PLA is rare. However, ATH was used for flame retardant PLA intended for durable end uses and for ease of recycling, where its depolymerization at high temperature (300 °C) was studied [30]. It was observed that the flame retardancy of PLA was because of physical effect and thus require relatively large quantities of ATH (50–65 wt %), which ended up affecting the mechanical properties of the polymer. In a similar development, combinations of ATH and phenolic resins were used as a flame retardant for PLA [31], and although the FR properties of the polymer improved, the crystal and mechanical properties were compromised due to high FR loading. To overcome the obvious challenge of poor mechanical properties associated with high ATH loading, the effect of ATH loadings (0, 10, 20, 30, and 50 wt %) with kenaf fibers (40 wt % loading) as primary reinforcement for PLA was investigated [32]. A 66% improvement in flame retardant efficiency and a significant enhancement in the storage modulus (136%) and tensile strength (59%) were obtained compared to the kenaf/PLA counterpart without ATH. Interestingly, it was concluded that the presence of ATH played a significant role not only as flame-retardant but also as secondary reinforcement to kenaf/PLA composites, which is at variance with the most popular assertion that high ATH loading compromises the mechanical properties of most polymers.

### 2.2. Phosphorus-Based Fillers

Phosphorus-containing FRs are among the most commercially viable flame retardants currently in use, but still under vigorous research due to their low loading, high efficiency, and the increasingly stringent flame-retardant requirements for plastics and textiles [33]. This review will not cover the entire research papers on phosphorus flame retardants because other researchers have reviewed them extensively [34,35,36]. This review is limited to recent applications of phosphorus flame-retardants for PLA.

**Scheme 1:**
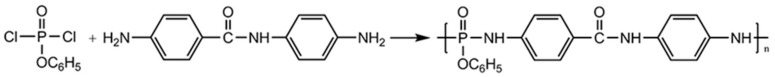
Polymeric phosphorus/nitrogen-containing FR.

Several phosphate-based FRs, such as ammonium polyphosphate (AP), melamine polyphosphate, aluminum phosphinate, and their combinations, have been used to introduce flame retardant properties to PLA [8]. In this regard, a polymeric flame retardant containing phosphorus and nitrogen was reactively synthesized through condensation polymerization and incorporated into PLA [37]. The structure of the nitrogen-containing phosphorus compound is shown in Scheme 1. The authors reported that a 30 wt % FR loading in PLA yielded an LOI of 25.5%, and UL-94 V-0 rating with 60% char residue in TGA tests in both air and nitrogen environments.

The efficiency of the polymeric flame retardant was attributed to the compact char formed on the surface of the composites during the burning process. The char served as a physical barrier which prevented the supply of oxygen [38], thereby protecting the bulk polymer from further degradation. The effect of the FR loading on the mechanical properties of PLA was, however, not studied. In another study, flame retardant polylactide was prepared with 25% FR loading to achieve UL94 V-0 rating with excellent anti-dripping properties using the combinatory effect of pentaerythritol phosphate, melamine phosphate, and polyhedral oligomeric silsesquioxanes [39]. It was suggested that the presence of polyhedral oligomeric silsesquioxanes in the composite was very instrumental in achieving the excellent anti-dripping effect. Although good FR PLA was achieved, the mechanical properties were compromised besides the problem of the high volume of smoke. Using the Mannich reaction, lignin was modified with formaldehyde and urea. The modified lignin was then combined with AP to produce intumescent flame retardant (IFR) [40]. The PLA/IFR composites had improved FR properties at 23 wt % loading with high char yield and improved thermal stability.

**Figure 1:**
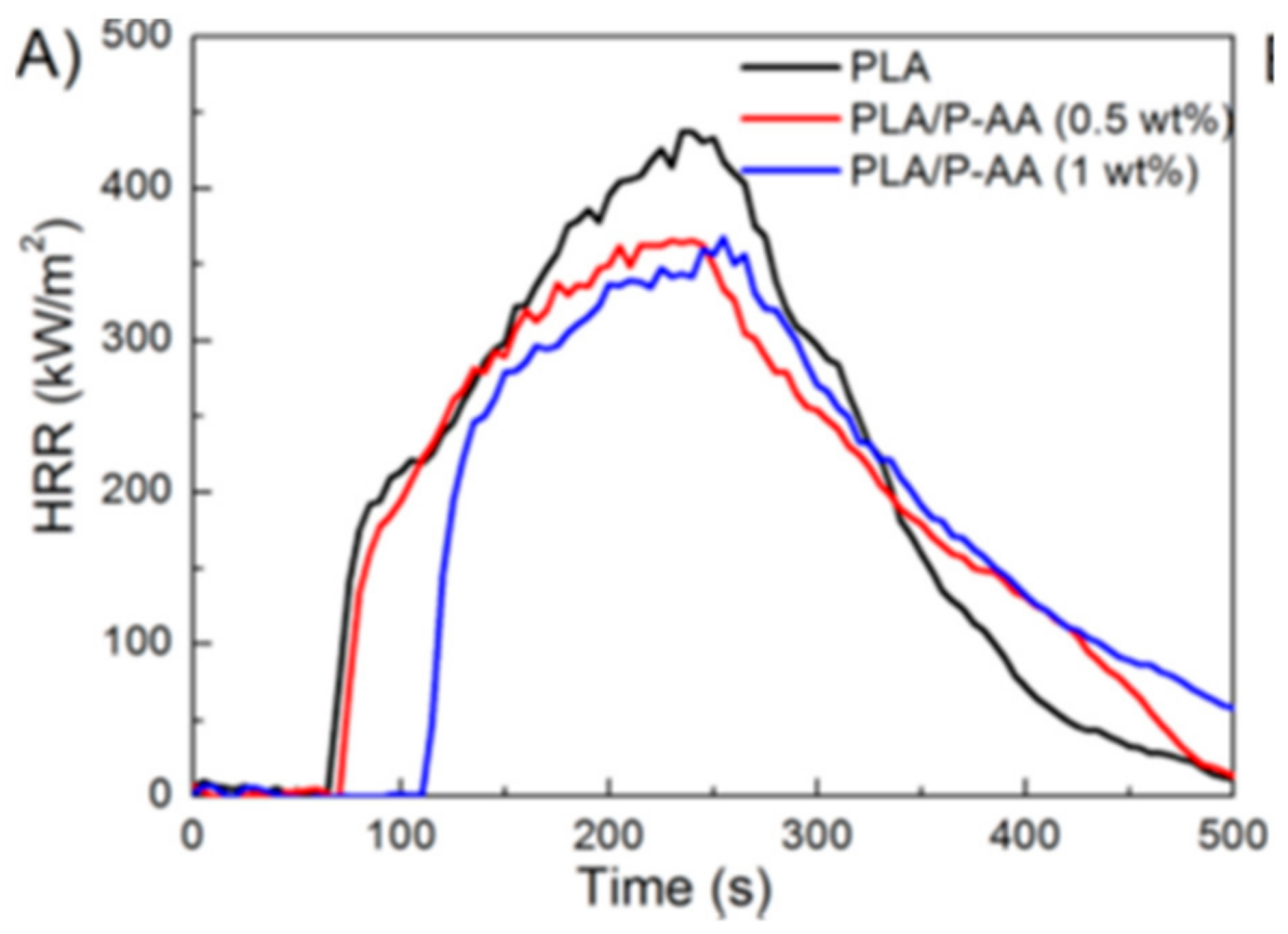
HRR vs. time curves of PLA and PLA/P-AA. (Adapted with permission from [42]).

The efficiency of commercial FR, Exolit AP has been investigated in PLA reinforced with different bio-based fillers that could act as char formers in the intumescent system but issues, such as melt dripping and high smoke content, could not be avoided [41]. To further improve this commercial product, both lignin and starch from different sources were added, which allowed the material to reach a UL-94 V-0 rating, while the non-reinforced material only achieved a V-2 rating. Meanwhile, the presence of starch and lignin in the composite increased the LOI alongside a significant reduction in PHRR. Despite the progress made in recent years using phosphorus/PLA FR formulations and their combinations, most of these success stories have not been commercialized possibly due to large volumes of toxic gases released during the pyrolysis process, which are usually not reported. There is also a lack of complete understanding of the FR mechanism. For instance, flame-retardant PLA based on gas−solid biphase N, N-diallyl-P-phenylphosphonicdiamide (P-AA) at 0.5 wt % loading was investigated [42] and an LOI of 28.4% with V-0 rating was attained. The biphase additive had an insignificant effect on the mechanical properties of PLA matrix with only a meager reduction in PHRR (347 kW/m^2^ to 366 kW/m^2^) (Figure 1).

**Figure 2:**
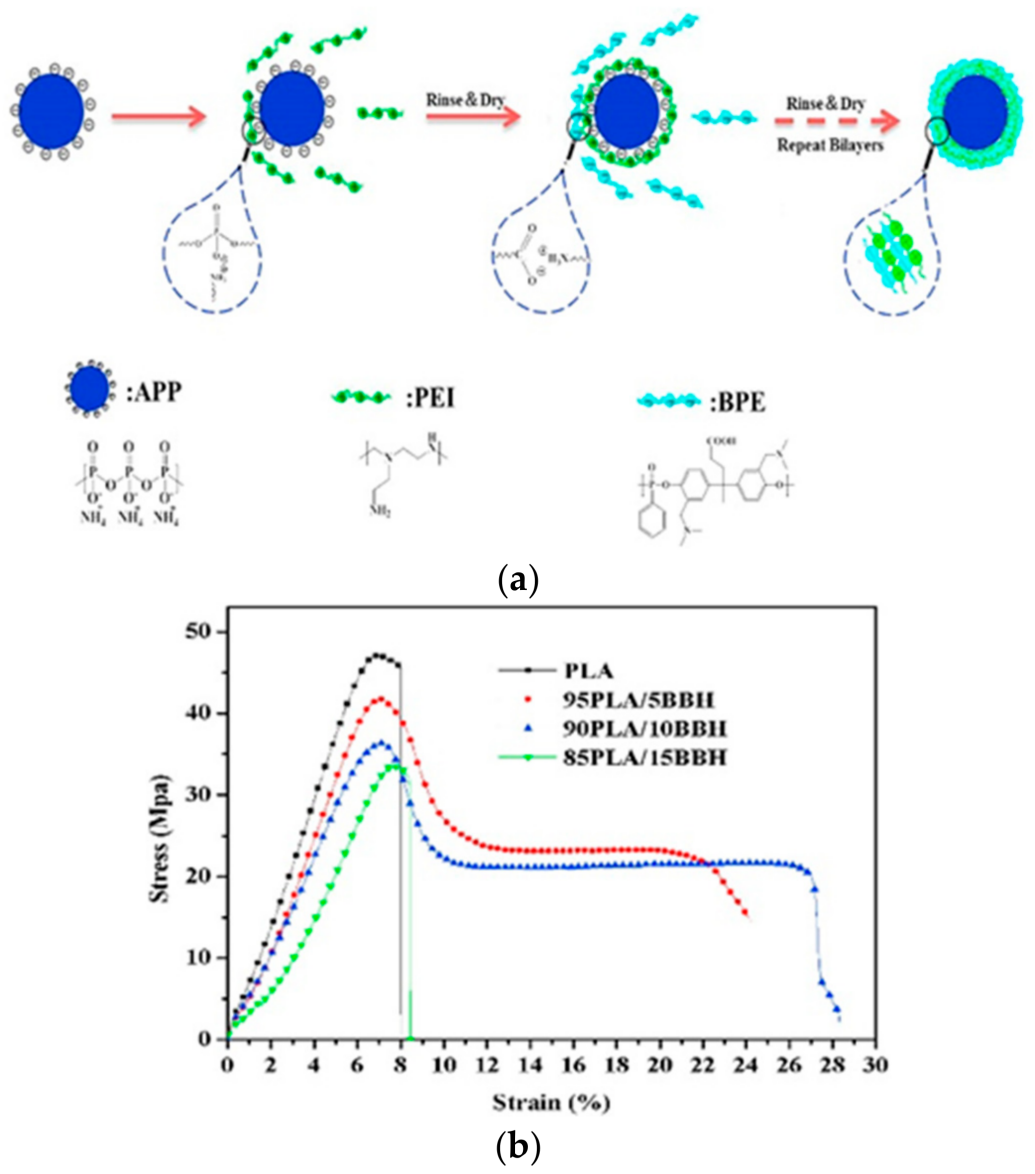
(**a**) Process for layer-by-layer assembly of FR; and (**b**) strain-stress curves of neat PLA and PLA/FR composites (Adapted with permission from [44]).

Recently, mechanically tough FR PLA was reported by reactive blending of PLA with ethylene-acrylic ester-glycidyl methacrylate terpolymer (EGMA), with the addition of 20 wt % aluminum hypophosphite (AHP) as an effective flame retardant [43]. The matrix achieved a V-0 rating with an LOI of 26.6% and an appreciable reduction in peak heat release with an increase in the elongation at break by 22%. Approximately 11% enhancement in the notched Izod impact strength was obtained compared to neat PLA. Meanwhile, as the content of AHP in the matrix increased, the tensile strength was affected, suggesting that a minimum balance ought to be maintained between FR loading and PLA to preserve its toughening properties. Similarly, a plant-derived diphenolic acid and inorganic core ammonium polyphosphate covered with organic shell via layer-by-layer assembly of polyelectrolyte and polyethyleneimine was investigated [44] (Figure 2a). This led to an enhancement in the FR properties and a significant improvement in the elongation at break of PLA composite, as indicated in Figure 2b. The FR PLA fabric achieved V-0 rating in the UL-94 at 10 wt % FR loading and 27.3% elongation at break compared to neat PLA. The improved mechanical property was attributed to the debonding and plastic void deformation of the PLA matrix around the FR [44]. 

Additionally, anhydrous manganese hypophosphite (A-MnHP) flame-retardant PLA composite at 15 wt % FR loading having increased tensile strength has been reported [45]. The increase in tensile strength of PLA/A-MnHP composite was attributed mainly to the unique morphology of A-MnHP in the matrix. Important FR efficiency indicators, such as PHRR, THR, peak CO_2_, and CO also reduced by 50%, 13%, 50%, and 53%, respectively, with an LOI value of 27.5%. Phosphorus-based flame retardants have an effectively less detrimental effect on the mechanical properties when applied alone in smaller quantities or in combination with other organic/inorganic flame retardants. Table 1 gives a complete overview of some of the typical phosphorus-based flame retardants and their combination with other FRs and their efficiency according to selected fire evaluation standards.

### 2.3. 2D Fillers

Two-dimensional materials are layered materials with strong in-plane chemical bonds and weak coupling between individual free-standing layers [62,63]. 2D materials have incredible properties because they are exceptionally strong, lightweight, and has high flexibility. This aspect of the review covers the various 2D nanomaterials applied as a flame retardant for PLA in recent years.

#### 2.3.1. Graphene

**Figure 3:**
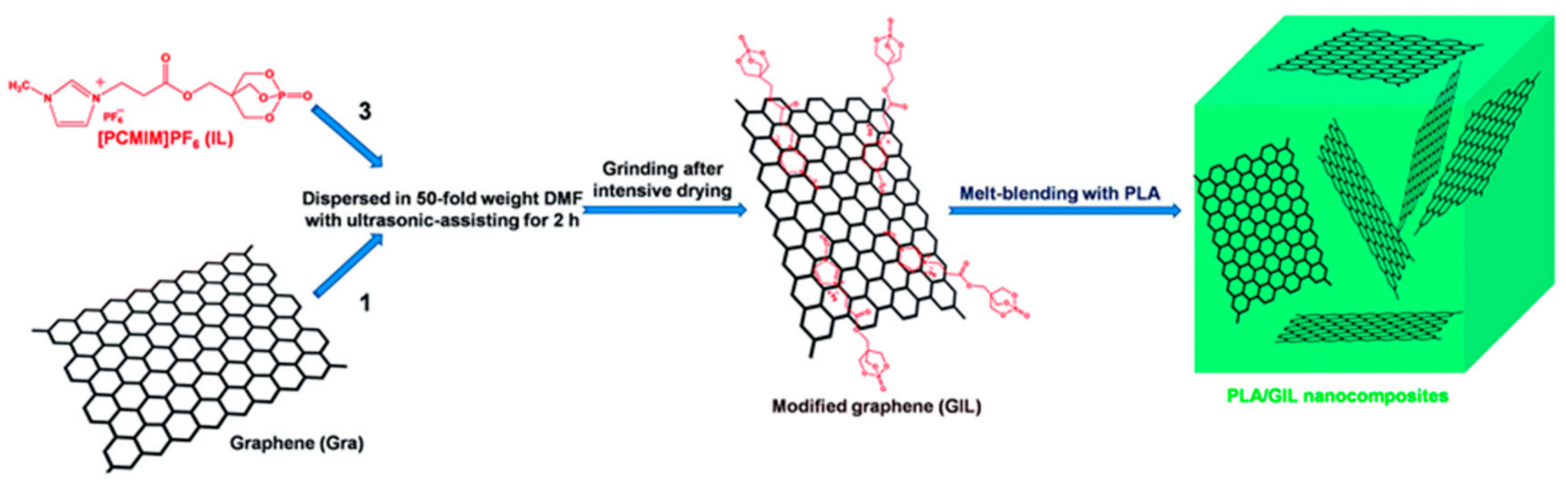
Surface functionalization of graphene using an ionic liquid (Adapted with permission from [66]).

The use of functionalized graphene oxide for flame-retardant PLA has been reported [64,65], although not very extensively. Typically, an ionic liquid containing phosphonium surface-functionalized graphene (GIL) was melt blended with PLA and its FR properties were evaluated [66]. It was realized that the fire-retardant performance of the PLA/GIL composites was significantly improved compared to either PLA/ionic liquid or PLA/graphene based on a reduction in PHRR and total heat released (THR). PLA/GIL showed an increased char yield compared to other composites. Figure 3 shows the surface functionalization process using phosphonium ionic liquid.

Although the mechanism for achieving flame retardancy has not been described, one can infer based on the information available that the GIL nanoparticles catalyzed the pyrolysis process leading to graphitization of the graphene oxide (GO) [67,68], which eventually functioned as a physical barrier to absorb degradation products. These were catalytically converted to a cohesive dense char on the surface of the polymer and inhibited the degradation products, heat, and oxygen transfer to protect the underlying polymer.

**Figure 4:**
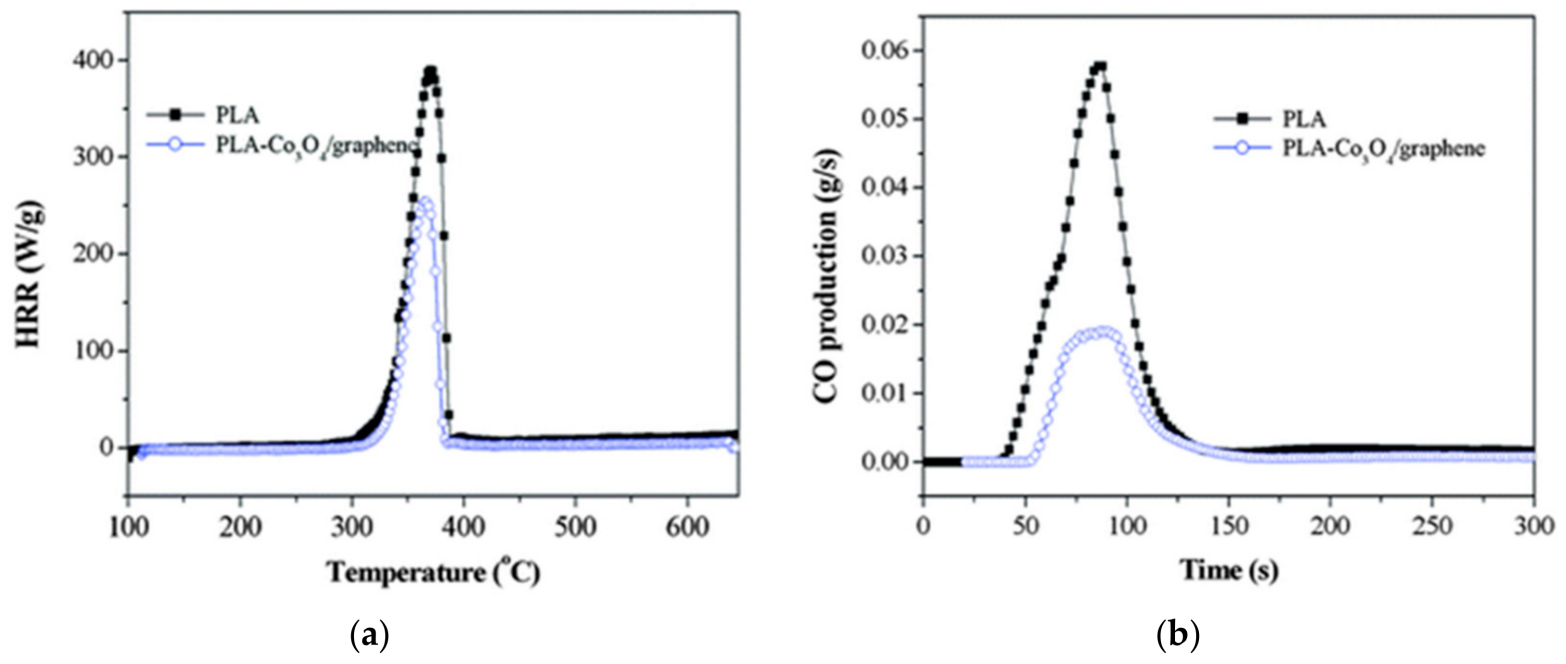
(**a**) Heat release rate and (**b**) CO curve of the PLA/CO_3_O_4_/graphene composite (Adapted with permission from [69]).

Similarly, PLA/CO_3_O_4_/graphene composite was prepared, and its FR and mechanical properties were investigated [69]. It was observed that the initial degradation temperature of PLA/CO_3_O_4_/graphene composite increased by 14 °C due to the inherent thermal stability of graphene. An approximately 40% reduction in PHRR was also attained, as shown in Figure 4a.

In addition, a significant decrease in gaseous products was obtained. The reduction in gaseous product was attributed to the barrier effect of graphene and delayed heat and mass transfer between the gas and condensed phase. Furthermore, most of the poisonous CO gas emitted from PLA during thermal decomposition was oxidized by CO_3_O_4_/graphene leading to a significant reduction (see Figure 4b). CO_3_O_4_/graphene FR provides a potential solution to reducing fire hazards associated with PLA. Their study, however, failed to investigate the effect of PLA/CO_3_O_4_/graphene composite on the mechanical and gas barrier properties of PLA. Conversely, the effect of graphene on the mechanical properties of PLA has been investigated [70], and the results showed that the presence of graphene in PLA greatly improved the tensile strength of the composites. Additionally, the melting temperature and the crystallization of PLA during the non-isothermal crystallization processes was enhanced. The improvement in mechanical properties was attributed to proper sonication of GO with the appropriate potential current and temperature, which separated the GO layers effectively, thereby enhancing the strength and ductility of the composite. It was observed that GO acted as an effective nucleating agent in enhancing the crystallization behavior of PLA. However, the presence of GO in the matrix resulted in 53% reduction in the transparency of PLA films. The study, however, failed to investigate the FR properties of GO/PLA composite.

#### 2.3.2. Clays

Nanoclays are nanoparticles of layered mineral silicates optimized for use in many fields. Polymer-clay nanocomposites are an especially well-researched class of nanomaterials due to their potential benefits, such as increased mechanical strength, decreased gas permeability, and superior flame-resistance [71,72]. Based on the chemical composition and nanoparticle morphology, nanoclays are organized into numerous classes, such as montmorillonite, halloysites, bentonite, kaolinite, and hectorite [73]. Organically-modified nanoclays (organoclays) are the attractive class of hybrid organic-inorganic nanomaterials with potential uses in polymer nanocomposites as rheological modifiers, gas absorbents, drug delivery carriers, and FRs [71]. In addition to the well-known FR chemistries, the use of nanoclay has been widely reported to improve the properties of PLA in addition to its good flame-resistance properties [72,73,74]. The incorporation of organo-modified nanoclay enhances the storage modulus of PLA in both solid and molten states, increases the biodegradability and flexural strength of the polymer in addition to its ability to decrease the heat release rate during the cone calorimeter test [75]. In a typical experiment, PLA/clay nanocomposites loaded with 3% organo-modified montmorillonite (O-MMT) and sodium montmorillonite (Na+ MMT) were prepared and their FR properties, molecular and supramolecular characteristics were studied using thermogravimetric analyzer (TGA), differential scanning calorimetry (DSC) and light microscopy (LM) [76]. It was concluded that the filler affected the ordering of the PLA matrix at the molecular and supramolecular levels but promoted char formation, reduced flammability, and enhanced its thermal stability. In a similar endeavor, the FR properties and melt stability of intumescent flame retardant (IFR) at 15 wt % and O-MMT at 5 wt % were studied [77]. The two systems created excellent flame retardancy, thus, achieving UL-94 V-O rating and an LOI of 27.5% with enhanced suppression of melt dripping. Similarly, a knitted fabric finished with PLA/clay nanocomposite in the presence of a plasticizer has been reported [78], in which a significant reduction in PHRR (38%) was attained. PLA-based nanocomposites were prepared using two different nanofillers: expanded graphite (EG) and organically-modified montmorillonite (O-MMT) were prepared and the thermal and mechanical properties were evaluated [79]. The improvement in thermal and mechanical properties obtained by the presence of both nanoparticles were associated with the good co-dispersion and the co-reinforcement effect by the EG. The enhanced FR property was attributed to the combined additive action of both nanofillers in the ternary system. No specific mechanism was suggested, but the FR properties were attributed to the formation of a ceramic/carbonaceous surface layer during the pyrolysis process. In another development, organo-modified layered silicates and β-calcium sulfate hemihydrate and β-anhydrite II were melt blended with PLA. At 43 wt % FR loading in PLA, the nanocomposite showed good dispersion, high thermal stability, and adequate mechanical resistance [80]. Cone calorimetry showed a significant increase in the ignition time compared to neat PLA and a substantial decrease (40%) in PHRR, whereas the UL-94 HB rating exhibited non-dripping with extensive char formation. It is important to state that while these fillers are presumed to be effective, some of the unique properties of PLA are lost due to high FR loading.

**Figure 5:**
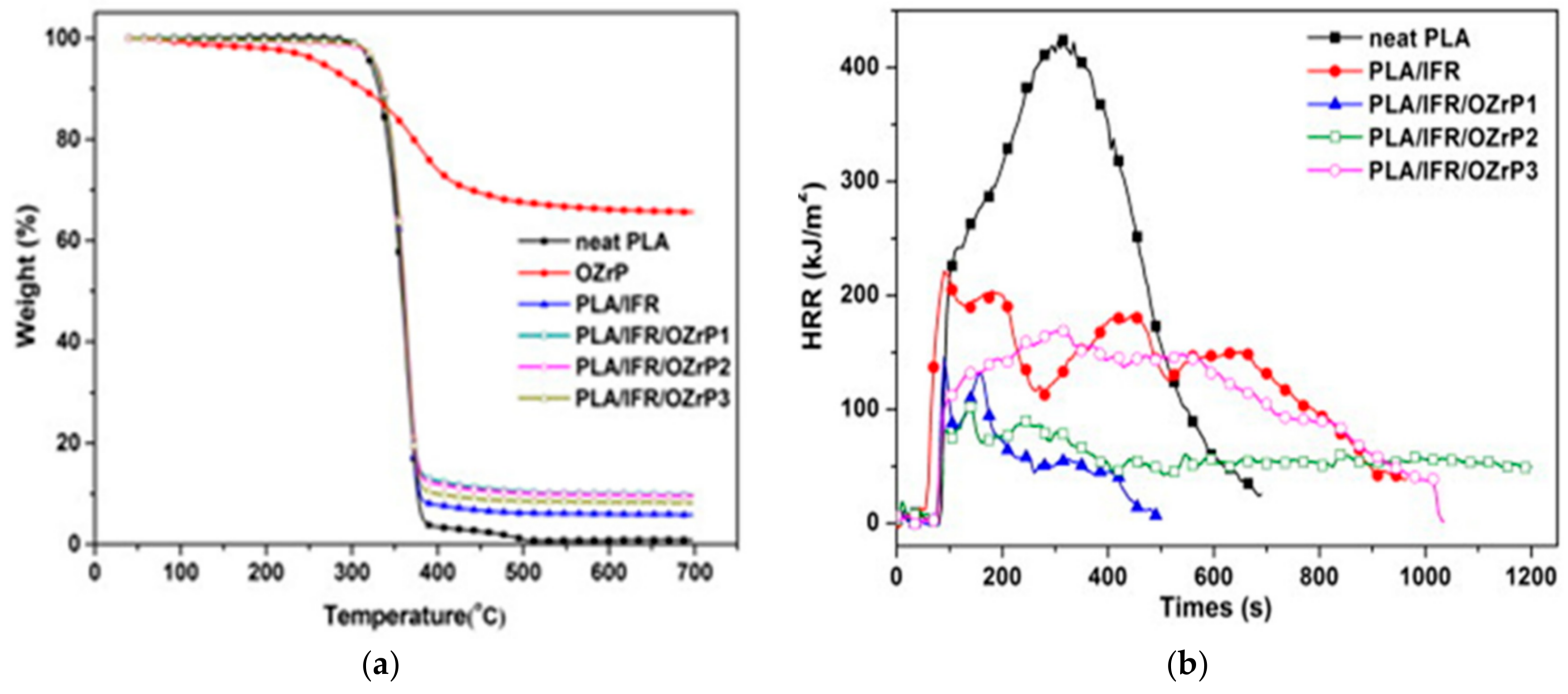
(**a**) TGA of PLA/IFR/α-ZrP composites; and (**b**) heat release rate of PLA/IFR/α-ZrP (Adapted with permission from [81]).

Αlpha-zirconium phosphate (α-ZrP) is an important layered nanostructure material because of its versatile nature and unique anion exchange properties. It has been widely applied as a FR for several polymers, but its application for PLA is not extensive. The combined effect of α-ZrP and intumescent flame retardant (IFR) on flame-retardant PLA was investigated [81]. The result suggests that α-ZrP increases the thermal stability and char yield of PLA (Figure 5a).

**Figure 6:**
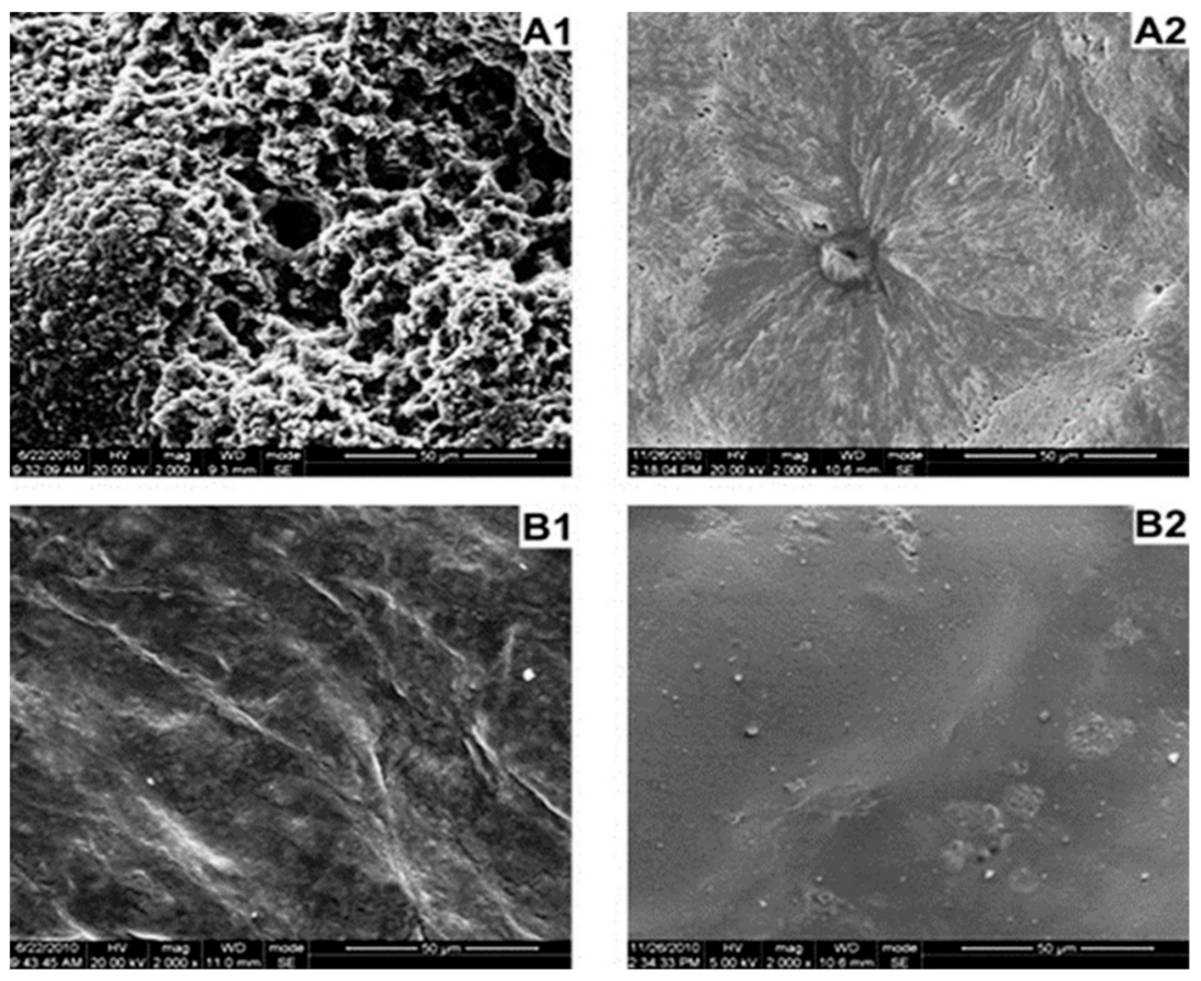
SEM images of char residue after cone calorimeter test: (**A1**,**A2**) the outer surface and the inner surface of PLA/IFR; and (**B1**,**B2**) the outer and inner surface of PLA/IFR/α-ZrP2 (Adapted with permission from [81].

Interestingly, 1.0 wt % of α-ZrP loading in addition to the intumescent flame retardant led to a 61% reduction in PHRR (Figure 5b), 81 wt % char residue after the cone calorimeter test, and an increase in LOI to 35.5%. The FR mechanism of the IFR/α-ZrP was attributed to the flawless compact, smooth, and tight char formed on the surface of the composite which served as a barrier to oxygen and other pyrolysis gases, as shown by the SEM images in Figure 6.

The firm, compact structure of the char obtained from the zirconium-doped PLA/IFR could no doubt resist mass and heat transfer and effectively retard the degradation of the underlying material. The smooth, homogeneous ceramic-like char obtained was attributed to the perfect balance between α-ZrP and the intumescent FR which protected the material throughout the combustion process and, thus, served as a mechanical reinforcement to the charred layer. This phenomenon reinforces the conclusion that alpha-zirconium phosphate is an efficient FR additive.

#### 2.3.3. Layered Double Hydroxides (LDH)

**Figure 7:**
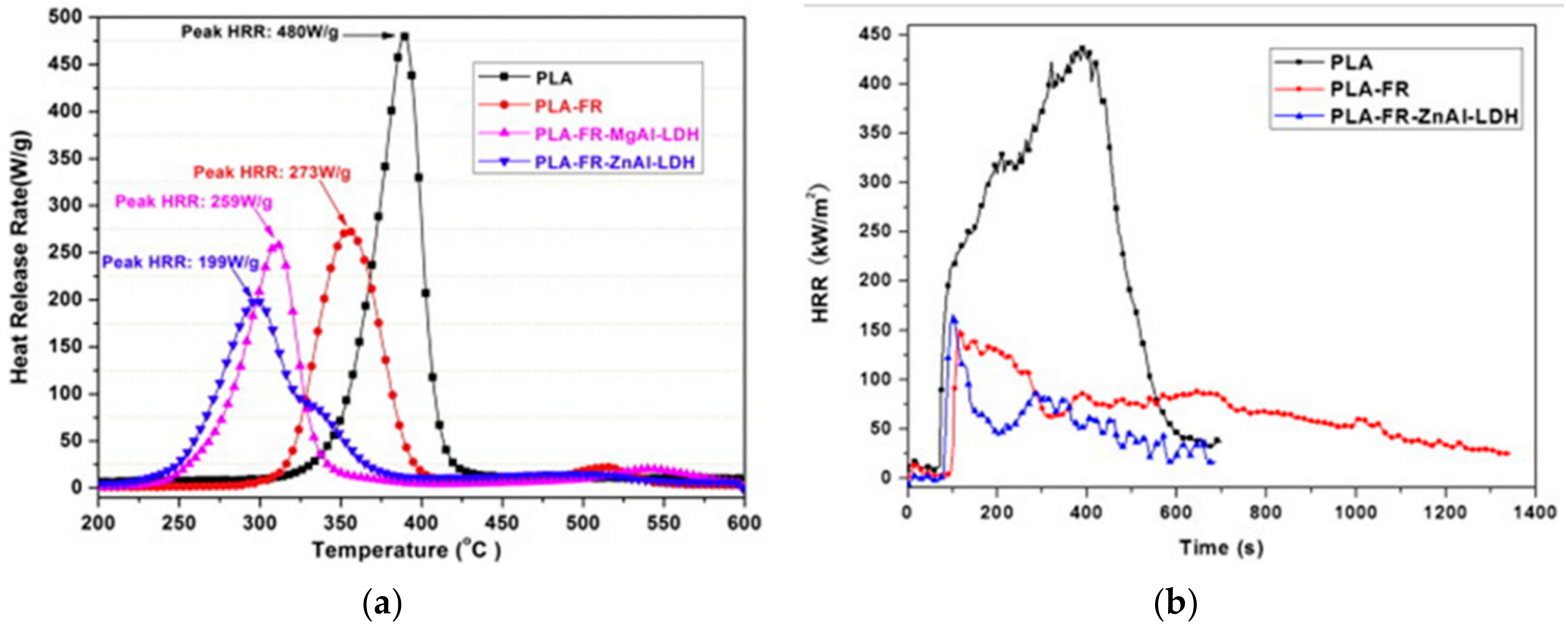
(**a**) Microscale combustion calorimeter PLA/FR/Zn-Al-LDH composite; and (**b**) HRR of PLA/FR/Zn-Al-LDH (Adapted with permission from [85].

Layered double hydroxides (LDHs) are an emerging class of flame retardants additives with great prospects in fire chemistry and smoke suppression due to their unique chemical conformations and layered structure [82,83]. The flame retardant and smoke suppression mechanism of LDHs involves the loss of interlayer water, intercalated anions, and dihydroxylation to form metal oxides, which absorb large amounts of heat and dilutes the concentration of O_2_ [84]. This phenomenon promotes the creation of carbonaceous char on the polymer thereby protecting the bulk polymer from air and suppressing smoke production due to suffocation. PLA nanocomposites based on organo-modified zinc aluminum layered double hydroxide (Zn-Al-LDH), ammonium polyphosphate (APP), pentaerythritol (PER), and melamine cyanurate (MC) in the mass ratio 1:2:2 at total loading of 25% was prepared [85], and the results showed that the presence of 2% Zn-Al-LDH in the PLA/FR matrix decreased the PHRR by 58% in both the microscale combustion calorimeter (MCC) and cone calorimeter (Figure 7a,b).

The FR mechanism of the composite is attributed to the heavy char formation, which slowed down the initial decomposition thereby reducing heat and fuel transfer in the burning process. The metal component (Zn-Al-LDH) in the form of nanoparticle catalyzed the reaction and, thus, led to massive graphitization. However, the consequence of Zn-Al-LDH on the mechanical properties of PLA was not assessed. Similarly, the effect of nickel-containing LDH and a cyclophosphazene compound, as well as sodium dodecyl sulfate (SDS) on the thermal stability and FR properties of PLA, were investigated [86]. It was observed that the Ni LDH-SDS with the various trivalent metals enhanced the LOI value and improved the UL-94 results by achieving a V-0 rating. Nevertheless, the tensile strength and elongation at break got compromised with increasing content of LDH. Correspondingly, a single macromolecular intumescent flame retardant synthesized by self-assembly of diethylenetriamine penta-(methylenephosphonic) acid with melamine (DT-M) and modified layered double hydroxides (LDH) by intercalating phytic acid (PA) into the LDH layers was investigated [87]. The PLA/intercalated modified LDH composites were prepared by the traditional melt blending technique and its FR properties were evaluated. From the results, 14 wt % DT-M and 1% PA–LDH increased the LOI value from 19.4% to 38.9%. Additionally, a V-0 rating was attained in the UL-94 test in addition to a significant decrease in the peak heat release rate by 62.9%. However, the mechanical properties (tensile strength, elongation at break) were slightly affected. To overcome the obvious mechanical defects associated with LDH-containing FRs on PLA, intumescent flame retardant (IFR) made up of silane-coated ammonium polyphosphate (APP), pentaerythritol phosphate (PEPA), and nickel aluminum layered double hydroxide (NiAl LDH) with cornstarch (CS) and poly(butylene succinate) (PBS) as mechanical reinforcers were investigated [88]. The PLA/PBS/APP/CS/NiAl LDH composites had excellent FR properties. The mechanical properties (i.e., tensile strength and elongation at break) of the PLA/PBS/APP/CS/NiAl LDH composites improved with increasing content of PBS and CS in the matrix. It is recommended that the content of LDH in the PLA polymer matrix be reduced in order not to compromise the mechanical properties for FR gain. Table 2 gives a summary of typical 2D nanomaterials and their combinations applied for flame-retardant PLA.

### 2.4. 1D Fillers

One-dimensional (1D) nanostructured materials, such as nanotubes, nanofibers, nanowires, nanoribbons, and nanobelts, have gained considerable research interest due to their remarkable properties and potential wide range of applications [97,98,99]. 1D nanomaterials have been applied in various fields such as electronics, magnetism, optics, and catalysis [100,101,102], and in recent times as FRs either alone or with other traditionally-known FR materials [103,104,105,106]. Research into 1D nanomaterials is constantly developing with new frontiers to expand their potential applications. The application of this novel material group as fillers for flame-retardant PLA has been reviewed in this section.

#### Carbon Nanotubes

**Scheme 2:**
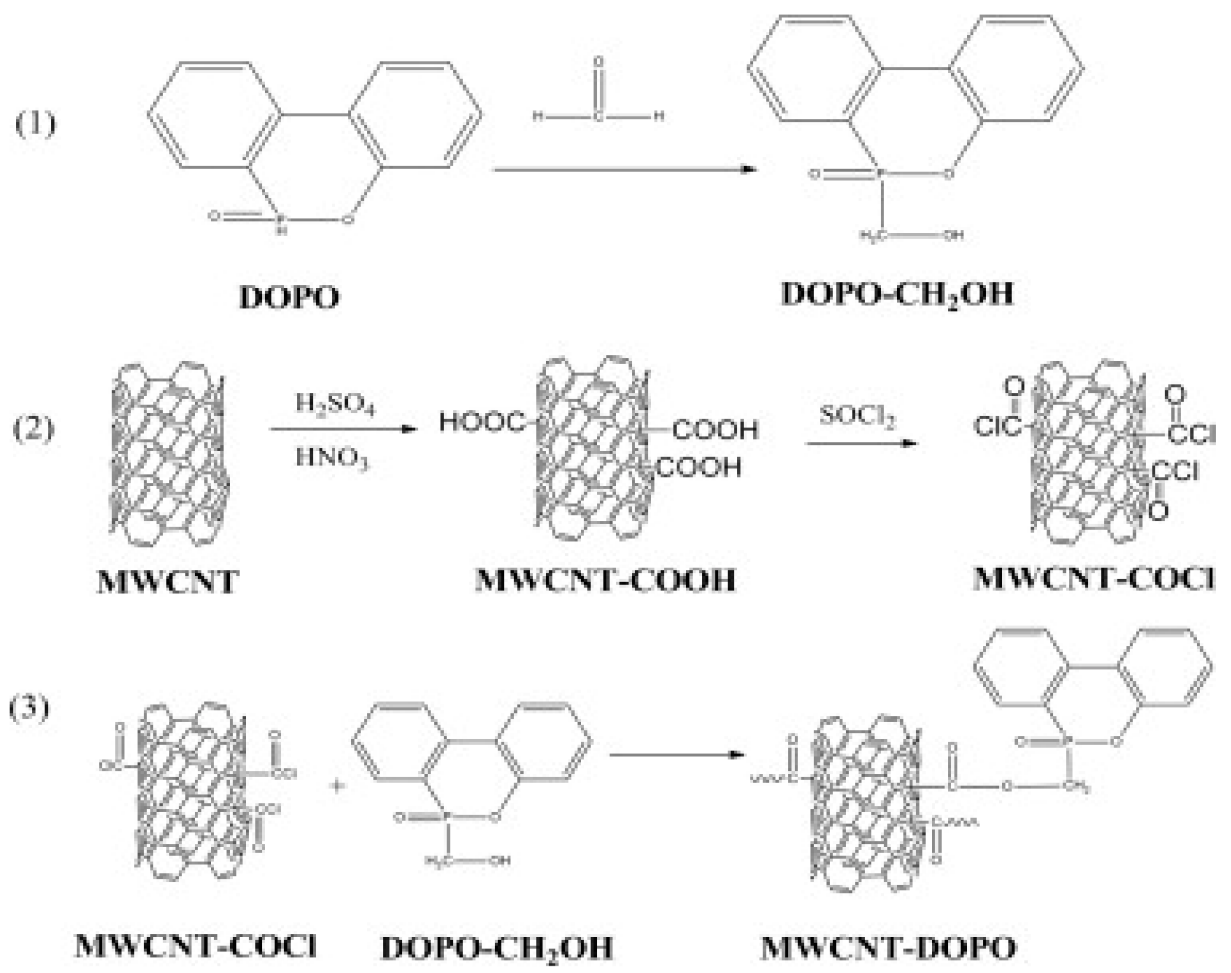
Synthetic process for functionalized MWCNT-DOPO (Adapted with permission from [111]).

Carbon nanotubes (CNTs) are allotropes of carbon with unique tubular structures of nanometer diameter and a large length to diameter ratio [104,105]. CNTs possess extraordinary thermal conductivity, high mechanical strength, and superior electrical properties. They have, therefore, found applications as additives to various structural materials due to their light weight, larger flexibility, and high thermal stability [106,107]. Nanodispersions of the various carbon nanotubes (single- and multi-walled carbon nanotubes) have been found to significantly improve the FR properties of diverse polymers [108,109]. CNTs can be used alone, modified, or combined with other materials/traditionally-known FRs. They can function as mechanical reinforcers for composites or purely as FRs due to their high thermal stability and char inducement effect. The high tensile strength and thermal stability of CNTs are attributed to the high basal-plane elastic modulus of graphite [107]. Based on these subtle properties, a PLA nanocomposite containing multi-walled carbon nanotubes (MWCNTs) was prepared by reactive extrusion and its FR properties were evaluated [110]. The results showed significant improvement in the flame spread, but a limited enhancement in PHRR. The insignificant reduction in PHRR was attributed to poor dispersion of the MWCNT in the PLA matrix. To achieve a significant reduction in PHRR or THR, MWCNT must be well dispersed within the matrix. Subsequently, the thermal, mechanical, and FR properties of functionalized MWCNT using 9,10-dihydro-9-oxa-10-phosphaphenanthrene 10-oxide (DOPO) [111]. It was observed that the presence of MWCNT-DOPO in ramie/PLA composites improved the mechanical properties marginally, whereas the thermal and FR properties were highly enhanced compared to virgin PLA. Meanwhile, the problem of melt dripping persisted. The MWCNT-DOPO modification process is illustrated in Scheme 2.

**Figure 8:**
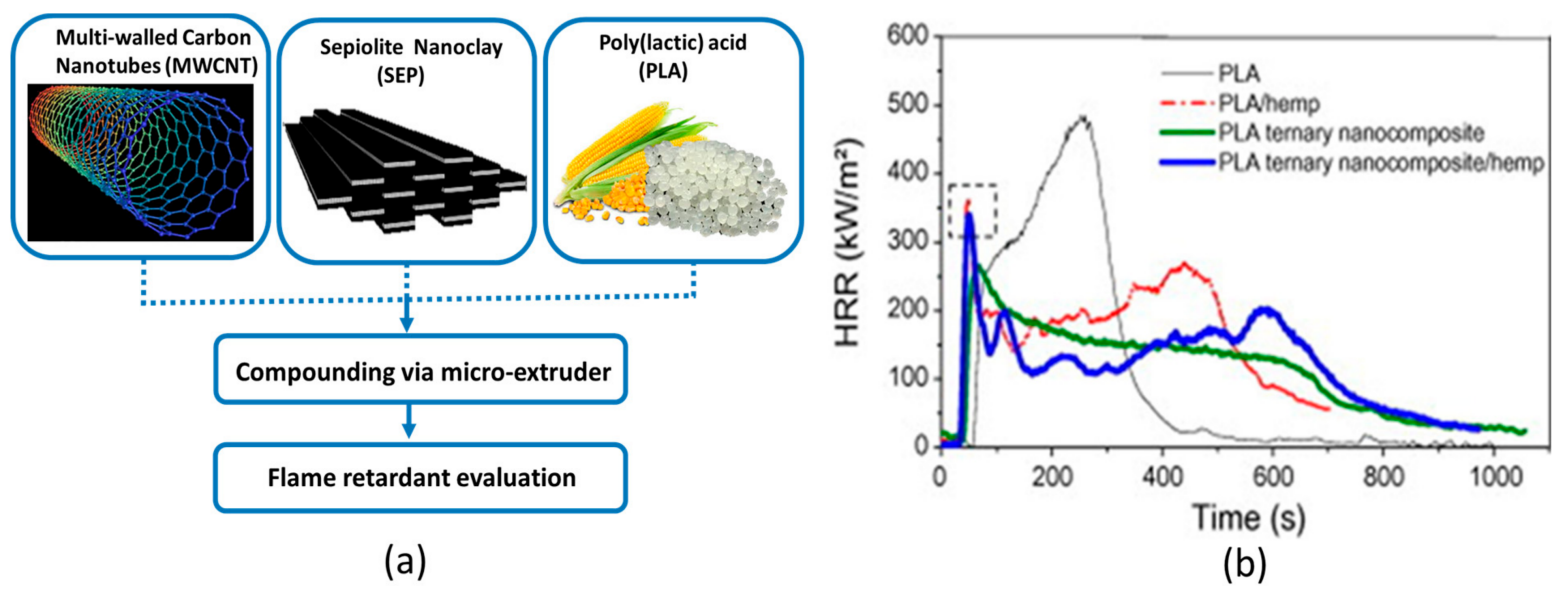
(**a**) Fabrication process of MWCNT/SEP/PLA composite (modified); and (**b**) PHRR of MWCNT/SEP/PLA composite (Adapted with permission from [96]).

A bio-based PLA composite with improved flame retardancy using sepiolite nanoclay and MWCNT (Figure 8) was proposed [96]. The composite was blended via micro-extrusion and its FR properties were evaluated. A 58% drop in PHRR at 20 wt % FR loading was attained using a mass ratio of 1:1 (MWCNTs: SEP). Meanwhile, the effect of this bio-based FR on the mechanical properties and smoke production rate of the composite was not reported.

Imidazolium phosphate (IP) functionalized MWCNT was blended with PLA in a mass ratio of 1:1 and the mechanical and FR properties were evaluated [112]. It was found that the presence of MWCNT increased the tensile strength and tensile moduli of PLA, whereas the elongation at break and impact strength decreased marginally. Also, the PLA/IP/MWCNT composite improved the fire safety of the composite due to obvious reductions in PHRR and THR. Although the FR mechanism was not suggested, it could be deduced that the IP catalyzed early char formation on the surface of MWCNT to ensure physical crosslinking, and thus resulted in a formidable compact char, which reduced the heat transfer and fuel in the burning process. The use of other 1D nanomaterials, such as nanowires, nanofibers for flame retardant PLA remains a field yet to be explored because a quick survey through the major scientific databases showed that these materials have not been researched extensively.

### 2.5. Polymer Molecules

#### Hyperbranched Polymers

Dendrimers and hyperbranched polymers are a class of macromolecules with unique physical and mechanical properties from the linear polymers and have several potential applications. Hyperbranched polymers have many reactive end groups, and low intrinsic viscosity (compared to linear polymers) and, usually, high solubility and miscibility with other materials. In comparison with dendrimers, they can be formed on a large scale in a few reaction steps, because their statistically branched structure is achieved in a single polymerization step [113].

**Figure 9:**
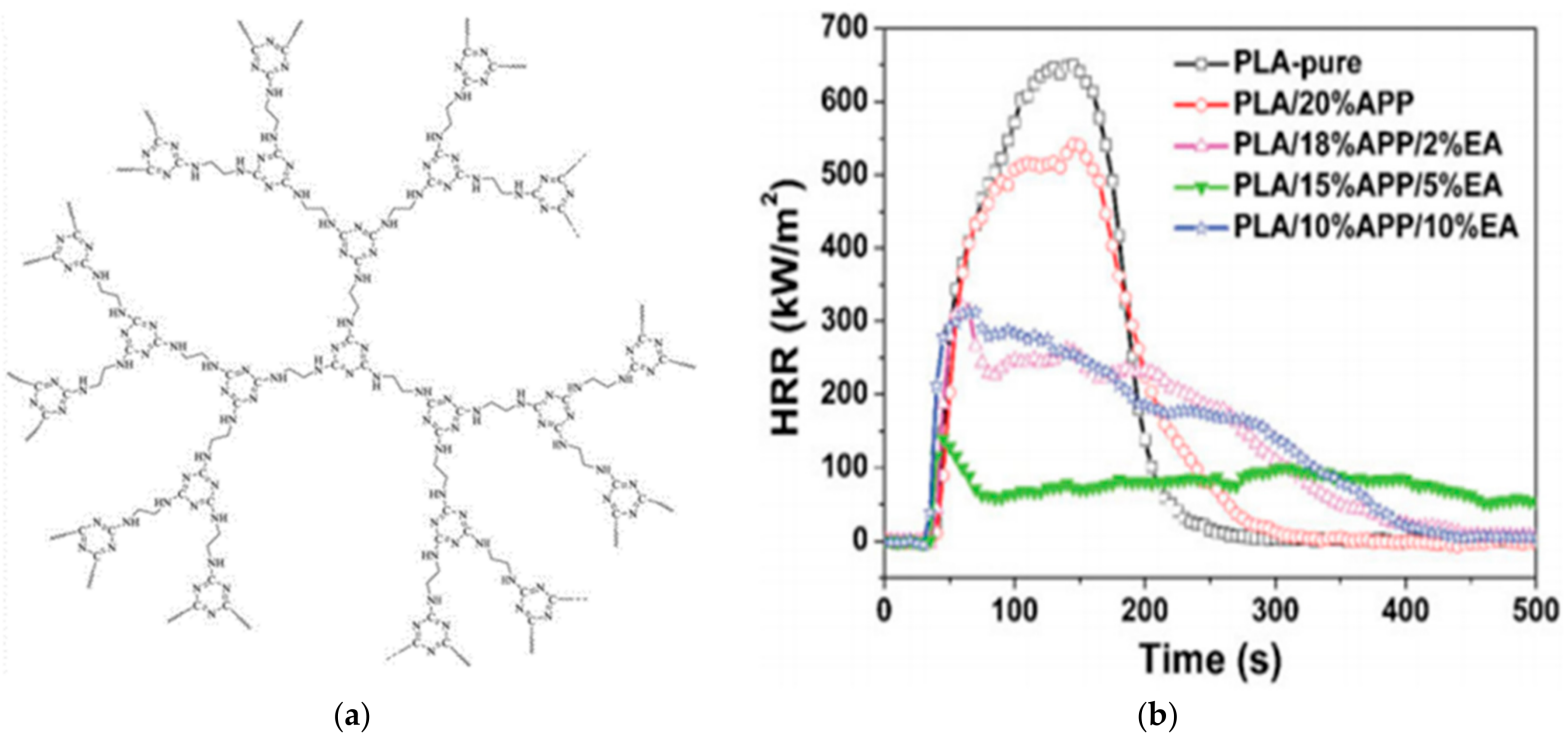
(**a**) Hyperbranched derivative of triazine (EA) and (**b**) PHRR /PLA/APP/EA (Adapted with permission from [117]).

The incorporation of hyperbranched polymers into PLA together with other traditionally-known flame retardants has been reported [114,115]. PLA composites containing a certain percentage of ATH and hyperbranched polymer (HBP6) via direct melt compounding was prepared where a high LOI value of 41% and UL-94 V-0 rating was attained with the mass ratio of 29:1 by ATH to HBP6 [116]. It was proposed that the presence of hyperbranched polymers in PLA/FR composites can improve its mechanical properties. Pursuant to this, the combinatory effect of a derivative hyperbranched polymer containing a triazine group (Figure 9a) and APP with PLA was studied in a mass ratio of 15:5 [117]. Unfortunately, its mechanical properties were not investigated, but the composite had excellent anti-dripping properties, high LOI value (41.2%), and lower PHRR (Figure 9b) compared to other hyperbranched flame retarded PLA composites. 

**Figure 10:**
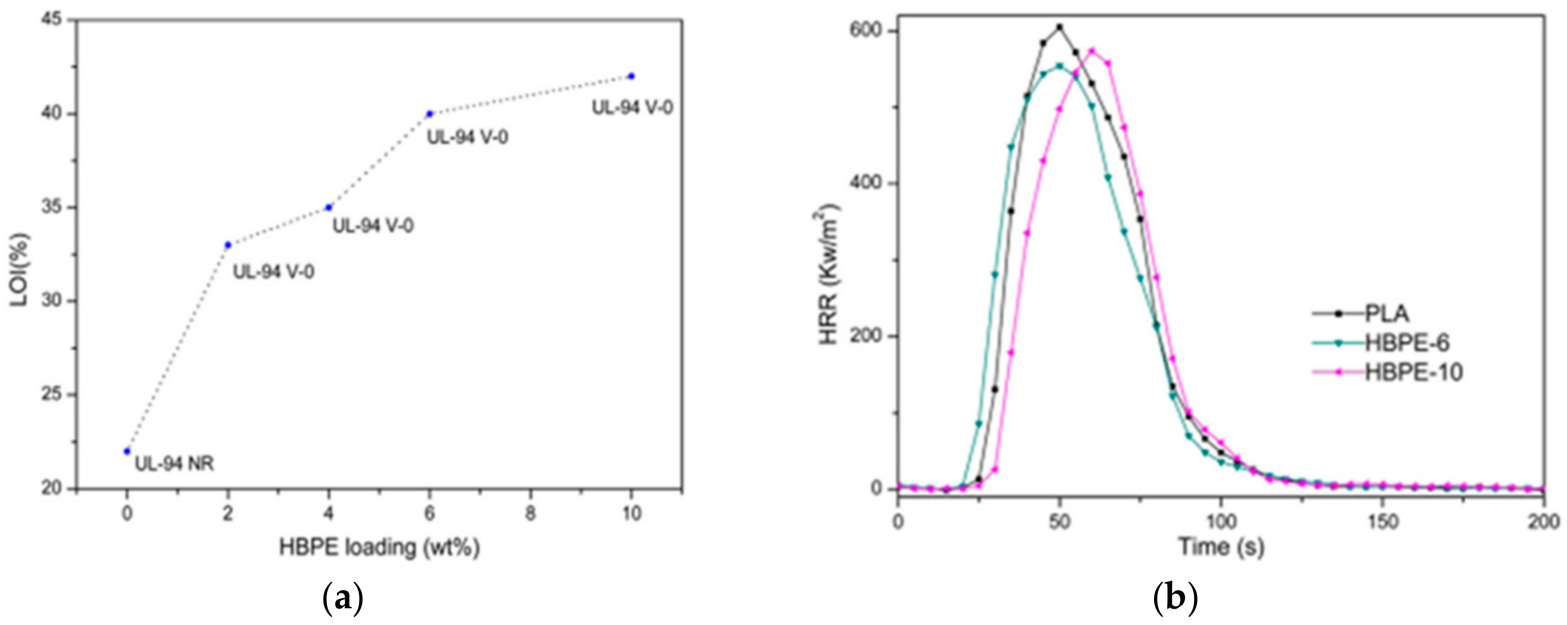
(**a**) LOI/UL-94 and (**b**) PHRR results of PLA/HBPE and composites (Adapted with permission from [119]).

Similarly, the effect of hyperbranched polyamine charring agent (HPCA) and APP on flame retardant and anti-dripping properties of PLA was studied [118]. It was observed that 30 wt % HPCA and APP loading at a mass ratio of 1:1 was ideal for obtaining excellent flame retardant and anti-dripping effects for PLA composites. Additionally, a hyperbranched poly(phosphamide ester) oligomer (HBPE) was synthesized via A_3_ + BB′ mechanism (3 parts of poly(phosphamide ester) oligomer and two parts of a derivative of triazine) and melt blended with PLA [119]. The FR properties of the HBPE/PLA composites were investigated using standard test procedures. Interestingly, only 2 wt % HBPE with PLA exhibited excellent flame retardancy (LOI value of 33% and UL-94 V-0 rating) (Figure 10a and as the content of HBPE increased to 10 wt %, the LOI value of the PLA composites also increased to 43% with a prolonged time to ignition (TTI) but with insignificant decrease (19.8%) in PHRR (Figure 10b). The minimal decrease in PHRR was accompanied by melt dripping and increased CO and CO_2_ emissions. The study, however, failed to evaluate the effect of HBPE flame retardant on the mechanical properties of PLA polymer.

## 3. Conclusions and Future Prospects

Flame-retardant PLA has received a great deal of attention due to its potential to replace petrochemically-engineered plastics, which have, over the years, posed serious environmental threats to biota. Additionally, the rather unpredictability oil prices and the fear of a possible shortage of petroleum deposits, new government policies restricting the use of hazardous plastics has and will ultimately spur the demand for flame-retardant PLA. This is evidenced by the large volume of research papers on flame-retardant PLA over the last decade. This trend is expected to continue because PLA has found major applications in many areas hitherto reserved for the so-called technical polymers. It is implicit from the papers reviewed that the metal oxide fillers have lost their attractiveness in flame-retardant PLA despite their significantly high efficiency in other polymers. This phenomenon can be attributed to their relatively high loadings and its consequential deleterious effect on the mechanical properties of PLA. However, the phosphorus-based FR PLA formulations have so far proven to be quite efficient, although most of the systems reviewed have not been commercialized. This class of FR has a higher prospect for the achievement of sustainable FR PLA composites. Among the 2D class additives, FRs made from modified nanoclays and their combinations with other traditionally-known FRs seems to have some commercial importance in terms of cost and ease of preparation in addition to their relatively high efficiency and minimal effect on mechanical properties. The remaining classes of 2D and 1D nanomaterials and their combinations are also effective, but their commercial viability remains in doubt possibly due to cost and ease of processing. Information available on the effect of these materials on the mechanical properties of PLA is not very consistent and, therefore, there is the need for a concerted effort to streamline issues in this regard. Although the use of polymeric molecules (including hyperbranched polymers) as flame retardants for PLA has not been investigated extensively, they still possess significant potential as efficient FRs. The combinations of the bio-based FRs with other compounds have great potential due to their efficiency and minimal effect on mechanical properties at low loading.

## Figures and Tables

**Table 1:** Phosphorus-based flame retardants and their combinations for PLA.

PLA Type/Brand and Supplier	Main FR Component	FR Loading wt %	LOI%	UL94	Cone Calorimetry Parameters (CCP)		TGA	Ref.
					PHRR Reduction %	*T*_5%_	Residue %/Max. *T* (°C)	
PLA nonwoven fabric from Shenzhen Shengdefu Cellulon Co. Ltd., China.	cyclic phosphonate ester	-	35	-	7 (A)	207.2	-	[46]
PLA (4032D) by Nature Works	Aluminum hypophosphite (AHP)	30	29.5	V-0	48.2 (C)	332	22.4	[47]
							(700 °C), Air	
PLA (4032D) (*Mw* 206,583 g/mo) by Nature Works	phosphorus-containing polymer or oligomer	13.32	23.0	V-2	4.7 (C)	415	40.9	[48]
							(700 °C), Air	
PLA from NatureWorks Ltd.	Organophophorus flame retardant	30	32	V-0	-	250	3.1	[49]
							(700 °C)	
							N_2_	
PLA (3052D) by Nature Works	Diphenolic biphosphate	16	27.4	V-0	19.62 (C)	342	7.51	[50]
							(700 °C), Air	
PLA (2002D) by NatureWorks	Poly(ethylene glycol) (PEG), Ammonium polyphosphate, boron phosphate, and tri-phenyl phosphate	32	32.4	V-0	-	295	2.8	[51]
							(700 °C), Air	
PLA (4032D) by NatureWorks	Silicone rubber composites and ammonium polyphosphate	20	29.2	V-0	42.1(C)	323	2.78	[52]
							(700 °C), Air	
PLA (2002D) by Natureworks	Tris(2-hydroxyethyl) isocyanurate polyphosphate melamine (TPM)	25	36.5	V-0	68 (C)	265	30.6	[53]
							(700 °C), Air	
PLA (3052D) by NatureWorks	Chitosan with ammonium polyphosphate (APP)	7	33.1	V-0	17.4 (C)	-	4.1	[54]
							(700 °C), Air	
PLA grade (6202D) by NatureWorks	Lignin and ammonium polyphosphate (AP)	20	-	V-0	32.3 (B)	320	14.3	[55]
							(700 °C), Air	
Polylactide by NatureWorks	Charring agent (CNCA-DA) containing triazine and benzene rings and APP	30	45.6	V-0	10 (C)	310	29.3	[56]
							(700 °C), Air	
PLA by NatureWorks	Lanthanum oxide (La_2_O_3_), Ammonium polyphosphate((APP), an Oligomeric charring agent with triazine (CNCA-DA)	24	42.7	V-0	27 (C)	316	4.2	[57]
							(700 °C), Air	
PLA (2002D from Cargill,)	POSS-modified nanofibrillated cellulose (PNFC), Ammonium polyphosphate (APP)	15	-	-	45 (C**)	-	-	[58]
PLA resin (4032D) by NatureWorks	Microencapsulated ammonium polyphosphate (APP) and melamine (2:1), Starch	30	41	V-0	76 (C)	25	22.2	[59]
							(600 °C),Air	
PLA resin (3051D) by NatureWorks	Phosphorus modified lignin {kraft lignin (P-K), organsolv lignin (P-O)	P-K 20	-	V-0	23 (C)	-	15	[60]
							13	
		P-O 20	-	V-0	33 (C)		(700 °C) Air	
PLA from NatureWorks supplied by Cargill	Starch, lignin, ammonium polyphosphate	40	32	V-0	50 (C)	-	-	[9]
PLA resin (3051D) by NatureWorks	Phosphorus, Microcrystalline and nano-crystalline cellulose (P-MCC &P-NCC), aluminum phytate (Al-PA)	P-MCC 20,	-	V-0	8 (C*)	-	-	[61]
		P-NCC 20		V-2	35 (C*)	-	-	
		Al-PA, P-MCC 20	-	V-2	38 (C*)	-	-	

Note: Sample type used for the cone calorimeter test: A denotes non-woven PLA, B is knitted PLA fabric, C is composite plate size 100 × 100 × 3 mm^3^, C* is composite plate size 100 × 100 × 4 mm^3^, C** is composite size 75 × 75 × 3 mm^3^.

**Table 2:** Typical 2D nanomaterials for flame-retardant PLA.

PLA Type/Brand and Supplier	Main FR Chemical Component	Total FR Loading/(LDH) %	LOI%	UL-94	Cone Calorimetry Parameters		TGA		Ref.
					PHRR Reduction %	TSR(m^2^/m^2^/Heat Flux (KWm^2^)	*T*-_5%_	Residue %/Max. *T* (°C)	
PLA (3052D) by NatureWo-rks	Diethylenetriaminepenta-(methylenephosp-honic) acid with melamine, and phytic acid modified LDH	15/(3)	38.9	V-0	63 (C)	0.81	300	10.66	[87]
								(700 °C)	
						35		N_2_	
PLA (2002D) by Polymer UNIC Technology (Suzhou) Co., Ltd.	NiAl LDH, Silane coated APP, pentaerythritol phosphate (PEPA), Corn starch, Poly(butylene succinate)	35/(2.5)	30	V-0	21 (C)	-	315	17.3	[88]
								(700 °C)	
						35		N_2_	
PLA (REVODE201) from Hisun biomaterials Co., Ltd. (Zhejiang, China	NiAl-LDH modified 2-carboxylethyl-phenyl-phosphinic acid	10/(10)	-	-	37 (A)	-	-	-	[89]
						35			
PLA (model 2002D) UNIC Technology Co., Ltd.	NiFe, NiAl, and NiCr LDH-SDS Hexaphenoxycycl-otriphosphazene (HPCP)	10/(2)	29	V-0	-	-	-	7.2	[86]
								(700 °C)	
								N_2_	
PLA Biomer L9000 from Biomer, Krailling, Germany	Zn-Al-LDH, ammonium polyphosphate pentaerythritol and melamine cyanurate	23/(2)	-	-	59 (C)	-	-	-	[85]
						35			
PLA (2003D) Nature-Works LLC	nano-clay, recycled bamboo chopstick fiber APP and expandable graphite (EG)	27/(6)	-	V-0	-	-	377.8	21.88	[90]
								(850 °C)	
								N_2_	
PLA (4042D) Natureworks	Hydrogel-organoclay	6/(3)	-	-	MCC	17.1	325	3.66	[91]
								(800)	
					28			N_2_	
PLA, (*Mw* = 150,000) Tong-Jie-Liang Biomaterial Co. Ltd.	Organically modified montmorillonite (OMMT) and aluminium diethylphosphinate (AlPi)	20/(5)	28	V-0	26.2 (C*)	-	328	4.87	[92]
								(700 °C)	
						35		N_2_	
PLA, (Ingeo 3051D) from NatureWorks, USA	OMMT, isopropylated triaryl phosphate ester	35/(5)	-	V-0	-	-	258.4	4.75	[93]
								(600)	
								N_2_	
PLA (2002D) by NatureWo-rks	OMMT, Aluminum trihydrate (ATH, H-42M grade)	50/(5)	42	V-0	65 (c***)	-	320	-	[94]
								(650)	
						50		Air	
PLA, (4032D) by Nature Works	Alpha-zirconium phosphate (α-ZrP)	(10)	26.5	V-0	42 (C***)	-	316	14.3	[80]
								(700 °C)	
						35		N_2_	
PLA, Galastic by Galactic S.A.	Organo-modified layered silicates (OMLS)	43/(3)	-	-	42 (C)	-	354	-	[95]
						35		(600) Air	
PLA (Biopearls) from Jongboom Holding B.V (Netherlands)	Sepiolite nanoclay and multi-wall nanotubes (MCWNT)	20/(10)	-	-	58 (C*)	-	336	N_2_	[96]
								13	
								(1000 °C), Air	
						35		N_2_	
PLA (4032D) by Nature-Works	Ionic liquid containing phosphonium and surface-functionalized graphene (GIL).	6/(4)	-	-	40 (C***)	-	331.4	9	[66]
								(800 °C)	
						35		N_2_	
PLA Cargill Dow Inc. (America)	Tricobalt tetraoxide-functionalized graphene composites (CO_3_O_4_/graphene)	1	-	-	40 (C**)	-	-	-	[69]
								(700 °C)	
						35		N_2_	
PLA by Galactic S.A.	Poly(ethyleneglycol) 1000 (PEG 1000) and montmorillonite, organo-modified	20/(3)	-	-	-	-	-	-	[74]
								(600 °C)	
								N_2_	
PLA from Dow–Cargill, Inc.	Organomodified particles of clay, unmodified particles of clay, plasticizer—Poly(ethylene glycol)	20/(3)	-	-	-	-	-	-	[76]
								(600 °C)	
								Air	
Tong-Jie-Liang Biomaterial Co. Ltd. (China)	Organically modified montmorillonite (O-MMT)	20/(5)	27.5	V-0	-	-	277	24.9	[77]
								(700 °C)	
								Air	
PLA Galastic, by Galactic S.A	Organomodified bentonite (Bentone^®^ 104-B104)	10	-	-	46 (B)	-	-	-	[78]
								(600 °C)	
						35		N_2_	
PLA (4032D) NatureWorks	Expanded graphite and organically modified montmorillonite	12/(3)	-		-	-	352	-	[79]
								(800 °C)	
								N_2_	

Note: MCC—microscale combustion calorimeter; Sample type used for the cone calorimeter test: A denotes non-woven PLA, B is knitted PLA fabric, C is composite plate size 100 × 100 × 3 mm^3^, C* is composite plate size 100 × 100 × 4 mm^3^, C** is composite size 100 × 100 × 3.2 mm^3^, C*** is 100 × 100 × 5 mm^3^.
